# Bone Marrow Mesenchymal Stromal Cells: Identification, Classification, and Differentiation

**DOI:** 10.3389/fcell.2021.787118

**Published:** 2022-01-03

**Authors:** Qianmin Gao, Lipeng Wang, Sicheng Wang, Biaotong Huang, Yingying Jing, Jiacan Su

**Affiliations:** ^1^ Institute of Translational Medicine, Shanghai University, Shanghai, China; ^2^ School of Medicine, Shanghai University, Shanghai, China; ^3^ School of Life Sciences, Shanghai University, Shanghai, China; ^4^ Shanghai University Institute of Advanced Interdisciplinary Materials Science, Shanghai, China; ^5^ Department of Orthopedics, Shanghai Zhongye Hospital, Shanghai, China; ^6^ Wenzhou Institute of Shanghai University, Wenzhou, China; ^7^ Department of Orthopedics Trauma, Shanghai Changhai Hospital, Naval Medical University, Shanghai, China

**Keywords:** bone marrow mesenchymal stromal cells, multipotent stem cells, skeletal stem cells, adipocyte lineage cells, BMSC

## Abstract

Bone marrow mesenchymal stromal cells (BMSCs), identified as pericytes comprising the hematopoietic niche, are a group of heterogeneous cells composed of multipotent stem cells, including osteochondral and adipocyte progenitors. Nevertheless, the identification and classification are still controversial, which limits their application. In recent years, by lineage tracing and single-cell sequencing, several new subgroups of BMSCs and their roles in normal physiological and pathological conditions have been clarified. Key regulators and mechanisms controlling the fate of BMSCs are being revealed. Cross-talk among subgroups of bone marrow mesenchymal cells has been demonstrated. In this review, we focus on recent advances in the identification and classification of BMSCs, which provides important implications for clinical applications.

## Introduction

BMSCs, first identified by Frieden (Friedenstein et al., 1976; Owen and Friedenstein, 1988), are a group of heterogeneous cells composed of multipotent stem cells, including osteochondral and adipocyte progenitors (Ashton et al., 1980; Pittenger et al., 1999; Wolock et al., 2019). BMSCs with niche forming and immunomodulatory ability are of great clinical significance and are widely explored in the treatment of autoimmune disorders and biological engineering (Orkin, 2000; Kong et al., 2009; Amini et al., 2012; Laranjeira et al., 2015).

Although the differentiation and hierarchies of hematopoietic stem cells have long been well clarified, those of BMSCs are less well defined (Azadniv et al., 2020), which limits BMSC application. Traditional RNA sequencing can only obtain the average data of cells, which fails to reflect cellular heterogeneity (Tang et al., 2019). The development of single-cell sequencing offers the opportunity to identify and classify BMSCs at the single-cell level. Through single-cell sequencing, several new BMSC subgroups have been identified, and their roles are clarified under normal physiological and pathological conditions.

In this review, we focus on recent advances in the identification and classification of BMSCs, key regulators, and mechanisms controlling BMSC fate and cross-talk among subgroups of BMSCs, to find important implications for clinical applications.

### Identification and Classification

**FIGURE 1:**
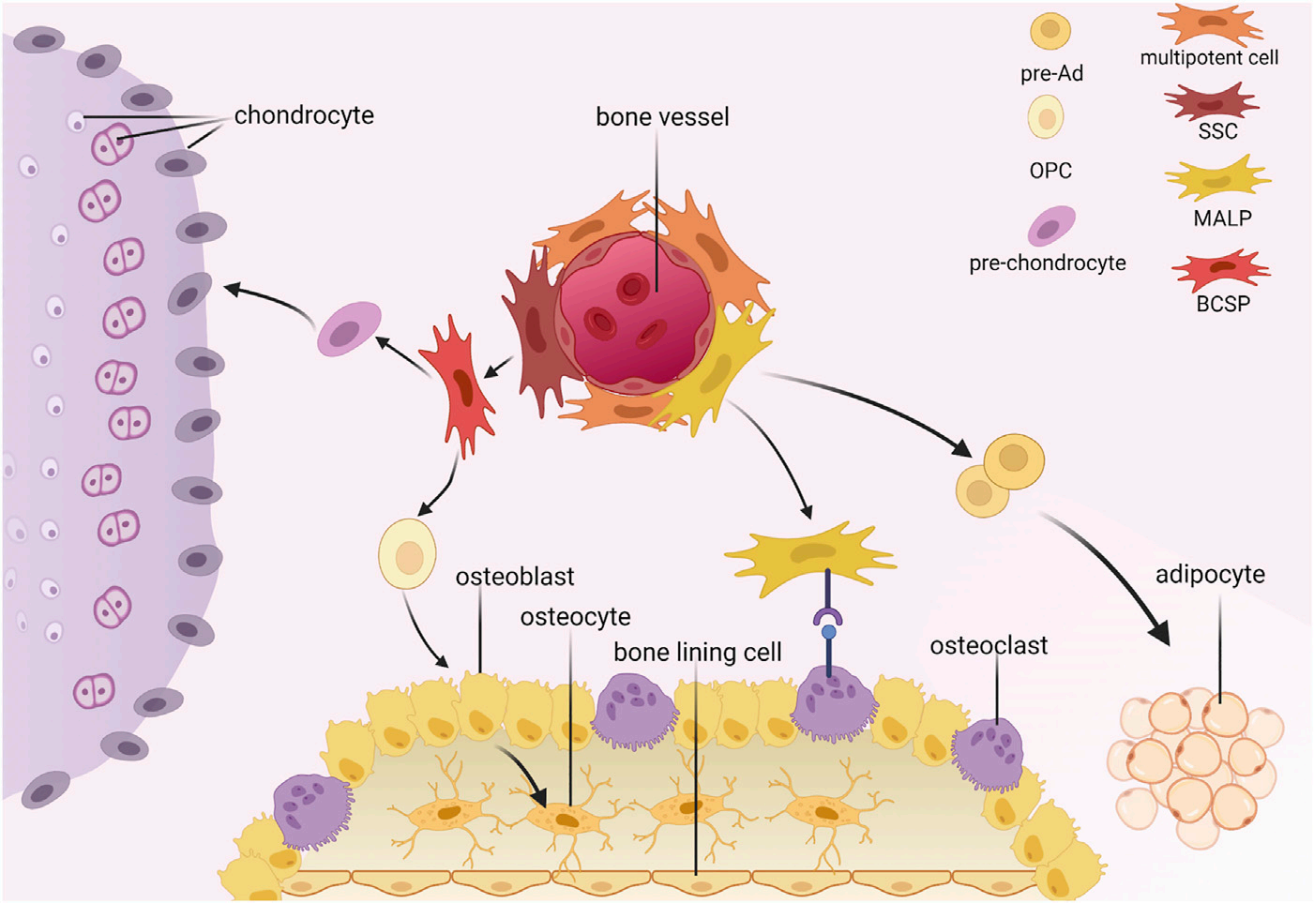
Hierarchy of bone marrow mesenchymal stromal cells. SSC, skeletal stem cell; MALP, marrow adipogenic lineage precursors; BCSP, bone, cartilage and stromal progenitor; pre-Ad, pre-adipocyte; OPC, osteo progenitor cell.

According to the International Society for Cellular Therapy, BMSCs are plastic-adherent when maintained in standard culture conditions and express CD105, CD73, and CD90 but not CD45, CD34, CD14, or CD11b, CD79α, or CD19 or HLA-DR surface molecules. In addition, BMSCs can differentiate into osteoblasts, adipocytes, and chondroblasts *in vitro* (Dominici et al., 2006). In this review, we emphasize the multipotent stem cells (MSCs), skeletal stem cells (SSCs), and adipocyte lineage cells (Figure 1).

### Multipotent Stem Cells

The identification of MSCs is mainly based on morphological observation (Li et al., 2016) and detection of surface markers (Baddoo et al., 2003; Ambrosi et al., 2017).

Novel molecular markers are continually being investigated to identify MSCs. LepR+ cells are a highly heterogeneous population containing multipotent stem cells. They are the main source of osteo-lineage cells and adipo-lineage cells in adult mice and generate the hematopoietic niche around bone vessels. Leptin receptor (LepR)+ cells are the primary source of multipotent stem cells that are stem cell factor (SCF)^high^ Cxcl12 ^high^ Nestin^low^ NG2^low^ (Zhou et al., 2014). Platelet-derived growth factor-α (PDGFRα)+ is another marker used individually or in combination for enriching MSCs (Suire et al., 2012; Grandl and Wolfrum, 2017). Its combinations include CD45-Ter119- (a nonhematopoietic marker) CD31-CD51+Sca1+ (Wolock et al., 2019), and CD45-Ter119-vascular cell adhesion molecule (VCAM) + CD146^low^CD31–PDGFRα+ (Chou et al., 2012). Glioma-associated oncogene (Gli1) +, a transcription factor and an effector of the Hedgehog pathways, enriches metaphysis mesenchymal progenitor cells (MMPs) located at the junction of cartilage just below the growth plate of postnatal mice. Hox11+ enriches MSCs in adult mice (Swinehart et al., 2013), especially in the periosteum and perivascular areas (Rux et al., 2016). In addition, CXCL12-abundant reticular (CAR) cells enrich bipotent progenitors of osteoblasts and adipocytes (Omatsu et al., 2010).

With the development of single-cell sequencing, we are able to further classify highly heterogeneous MSCs (Tikhonova et al., 2019). In addition, with a single trajectory, we can further characterize the hierarchy and differentiation routes of osteoblasts, chondrocytes, and adipocytes (Wolock et al., 2019). Wolock assigned cell state labels to each cluster of the scRNA-seq dataset and inferred the gene expression trajectories of MSCs isolated by flow cytometry. In this study, mesenchymal stromal cells represented the starting states and the most abundant population in the dataset. Gene sets of extracellular matrices, BMP2 targets, adipose tissue stromal cells, and HSC-supportive stromal cell lines were enriched in mesenchymal stromal cells, including B3galnt1, Cebpa, Cxcl12, Cybb, Il7, Kitl, Lpl, and Snai2. Mesenchymal stromal cells differentiate into adipocyte progenitors and osteoblast-chondrocyte progenitors. The second layer of hierarchy consists of adipocyte progenitors (AdPs) and osteoblast-chondrocyte progenitors (OsPs). The third group consists of preadipocytes (pre-Ad) and preosteoblast chondrocytes (Pre-OCs). Gene set enrichment analysis of TNFA signaling *via* NFKB showed that during adipogenesis, adult tissue stem cells are enriched in AdPs and pre-Ad, including Adipoq, Ccl2, Cebpb, Cxcl12, Fos, Il6, Jun, and Kitl. OsPs and pre-OCs were enriched in the gene sets of extracellular matrices, epithelial–mesenchymal transition, extracellular space, and tissue development, including Alpl, Mmp13, Poxstn, Sp7, and Wif1. Pro-osteoblasts and prochondrocytes served as end states of this dataset. Pro-osteoblasts were enriched in the gene set of endoplasmic reticula, Golgi apparatus, skeletal system development, and ossification, including Bglap, Col1a1, Col1a2, Creb3l1, Mef2c, Nupr1, Spare, and Sp7. Prochondrocytes were enriched in tissue development, extracellular space, and biomineral tissue development genes, including Ackr3, Ank, Cd44, Dmp1, Mepa, Mmp13 Nupr1, and Spp1 (Wolock et al., 2019).

Tikhonova classified LepR+ cells into four clusters by scRNA-seq: adipogenesis-associated clusters were P1 (Mgp high) and P2 (Lpl high), suggesting a poised pro-adipogenic state; clusters P3 (Wif1 high) and P4 (Spp1 high Ibsp high) represented osteoprimed LEPR+ cells (Tikhonova et al., 2019).

Zhong and others subdivided MSCs. They further classified LepR+ cells and reported the following hierarchy of LepR+ cells: early mesenchymal progenitors (EMPs) as the state status (highly expressing the genes Ly6a, Cd34, Thy1, Mfap5, Gsn, and Cles3b); intermediate mesenchymal progenitors (IMPs) (expressing higher levels of osteogenic genes than EMPs and located after EMPs); late mesenchymal progenitors (LMPs) (highly expressing the genes Aspn, Edil3, Tnn, Pstn, Ostn, and Dkk3); lineage committed progenitors (LCPs); and three final states of osteoblast/osteocyte (highly expressing the genes Sp7, Runx3, Col1a1, Ibsp, Bglap2, and Dmp1), adipocyte (highly expressing the genes Cebpa, Cebpb, Pparg, Lpl, Adipoq, and Apoe), and chondrocyte clusters (highly expressing the genes Sox9, Col2a1, Col10a1, Pth1r, Acan, and Ihh) (Zhong et al., 2020).

In the studies mentioned above, some functional features of MSCs have arisen. LepR+ MSCs are located around sinusoids and arterioles, are a main source of adipocytes in adult bone marrow (Tikhonova et al., 2019), and are regulated by the Pten gene to promote osteogenesis and to restrain adipogenesis (Zhou et al., 2014). CAR LepR+ MSCs can retain HSCs and colony-forming progenitors. During growth, Hox11 + can form perichondrium, tendons, and muscle connective tissue (Swinehart et al., 2013). Under irradiation, LepR+ MSCs can form osteoblasts and adipocytes (Zhou et al., 2014).

When discussing MSCs, we emphasize their potential for differentiation in multiple directions. The starting point of the branch of BMSCs has long been discussed, yet there is still no common resolution. After assembling the current studies that identify and classify MSCs through various markers or their combination, we tried to illustrate some functional relations and provide some implications for clinical applications.

### Skeletal Stem Cells

SSCs were once thought to be equal to BMSCs (Li et al., 2016) and to functionally give rise not only to osteoblasts and chondrocytes but also to adipocytes (Robey and Riminucci, 2020). However, recent studies have determined that SSCs, as a lineage-restricted subset of BMSCs, are especially characterized by self-renewal and osteochondral (Méndez-Ferrer et al., 2010; Chan et al., 2013; Marecic et al., 2015a; Worthley et al., 2015) differentiation (Méndez-Ferrer et al., 2010; Chan et al., 2013; Marecic et al., 2015a; Worthley et al., 2015).

Various studies have identified and defined different SSCs by detecting different sites of active bone growth. Therefore, it may be easier to understand SSCs from a clinical and functional perspective.

**FIGURE 2:**
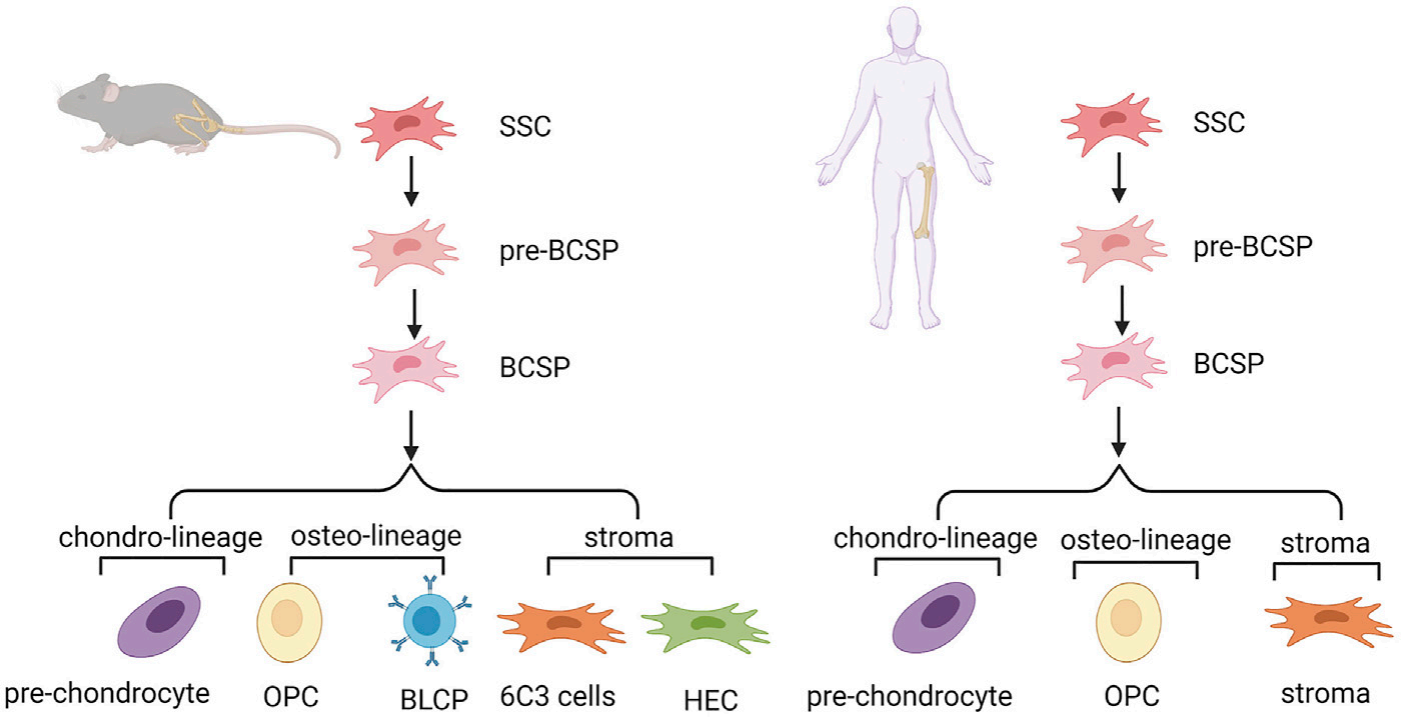
Hierarchy of skeletal stem cells in mouse and human growth plate. SSC, skeletal stem cell; pre-BCSP, pre-bone, cartilage and stromal progenitor; BCSP, bone, cartilage and stromal progenitor; BLSP, B-cell lymphocyte stroma progenitor; OPC, osteo progenitor cell.

In the mouse growth plate, the system of SSCs and their downstream progenitors can be described as lineage hierarchy. Determination of the mouse SSC (mSSC) lineage hierarchy included mSSCs and pre-mBCSP cells (CD45-TER119-TIE2-ITGAV+THY1-6C3-CD200-CD105-). Then, mBCSP cells (CD45-TER119-TIE2-ITGAV+THY1-6C3-CD105+) were generated, followed by chondro-lineage/PCP cells (CD45-TER119-TIE2-ITGAV+THY1+6C3-CD200 + CD105+), osteo-lineage cells, and stroma cells. The osteo-lineage can be further classified into two subgroups, namely, THY (CD45-TER119-TIE2-ITGAV+THY1+6C3-CD200-CD105+) and B-cell lymphocyte stroma progenitors (BLSPs) (CD45-TER119-TIE2-ITGAV+THY1+6C3-CD105-). The stroma can also be further classified into two subgroups, that is, 6C3 (CD45-TER119-TIE2-ITGAV+THY1-6C3+CD105+) and hepatic leukemia factor-expressing HECs (CD45-TER119-TIE2-ITGAV+THY1-6C3+CD105-) (Chan et al., 2015). The same group also reported on SSCs in human tissue. Nonhematopoietic SSCs can be prospectively isolated and characterized by PDPN+CD146-CD73+CD164+ (Chan et al., 2018). Another study isolated an intermediate cell type (CD200+ CD105-) between the start cluster mSSCs and BCSPs. These cells are self-renewing, pluripotent, and give rise to all other cells at the single-cell level. Pre-mBCSPs and mSSCs are functionally indistinguishable, so this population may be collectively referred to as phenotypic mSSCs (Gulati et al., 2018). PTHrP+ resting chondrocytes were identified as SSCs in the stationary region of the growth plate partially overlapping with mouse skeletal stem cells and progenitor cells previously identified by Chan and others. These cells are distributed in the perichondrium during the fetal stage (Mizuhashi et al., 2018) (Figure 2).

A study reported the existence of an SSC pool within the periosteum. Compared with bone marrow SSCs, periosteum SSCs are more clonal and have stronger growth and bone regeneration ability (Allen et al., 2004). Ctsk was found to label a type of SSC called periosteum stem cells that exist in the periosteum mesenchyme of long bones or the skull (Debnath et al., 2018). Gli1+ cells are osteogenic, chondrogenic, and weakly adipogenic cells in the sutura cranii (Zhao et al., 2015; Farmer et al., 2021). Axin2+ can label osteogenic and cartilaginous cells in the cranii (Maruyama et al., 2016).

In the hematopoietic microenvironment in human bone marrow, Sacchetti found a cluster of CD146+TIE2- cells around sinusoids defined as SSCs (Sacchetti et al., 2007). Another study from the same group illustrated that CD146 is able to functionally regenerate bone and stroma and to form a homotopic niche (Serafini et al., 2014).

As shown above, we can see that the tight relationship between active bone generation and the location of SSCs was vividly shown above.

### Adipocyte Lineage Cells

Adipocyte lineage cells represent a branch of BMSCs after the differentiation point in the pseudotime trajectory according to several studies.

Berry and Rodeheffer described adipocyte lineage cells consisting of CD24+ cells (generating adipocyte progenitors) and CD24^−^cells (generating preadipocytes) (Berry and Rodeheffer, 2013). Gupta reported the use of the transcription factor zinc-finger protein (Zfp)423 to enrich preadipocytes in bone marrow, which is generally used to label adipogenic cells in white adipose tissue (WAT) (Gupta et al., 2012). In addition, Zfp423 is able to distinguish preadipocytes from adipogenic progenitor cells in bone marrow. According to Ambrosi’s study, marker combination adipogenic progenitor cells can be enriched in CD45-CD31-Sca1+Pα+CD24-Zfp423- and pre-Ads by CD45-CD31-Sca1-CD24-Zfp423+ (Ambrosi et al., 2017). Another study mentioned above identified adipocyte lineage cells of LepR+ cells. Tikhonova reported but did not clearly define two adipogenesis-associated clusters P1 (Mgp high) and P2 (Lpl high), which presented a continuous relationship among four subsets of LepR+ cells. They were specifically found covering the sinusoidal capillaries as LepR+Esm1+ cells (Tikhonova et al., 2019). However, the association between these two has not been fully developed (Tencerova and Kassem, 2016).

There were significant differences in cell size and fatty acid content between intramedullary and extramedullary fat cells. Zhong first classified adipocyte lineage cells from a morphological perspective. There was a novel population of adipocyte lineage cells containing no lipid droplets in the cytoplasm. Zhong further identified a novel population that does not express Plin1, a lipid droplet coating protein gene, but does express Pparg, Cebpa, Adipoq, Apoe, Lpl, Lepr, Cxcl12, Il1rn, Serpina3g, Kng1, Kng2, Agt, Esm1, and Gdpd2 *in vivo* and named them marrow adipogenic lineage precursors (MALPs). In conclusion, MALPs express adipocyte markers but do not contain lipid droplets. As nonproliferative precursors of adipocytes, they are abundant in the form of pericytes and stromal cells, forming ubiquitous 3D networks in the bone marrow cavity, maintaining the bone marrow vascular system and inhibiting bone formation (Zhong et al., 2020). However, the pericyte function of these cells is not totally clear. The MALP knockout experiment did not change anything with respect to hematopoiesis. Zhou claimed that bone marrow adipocytes secrete SCF to promote the regeneration of stem cells and hematopoiesis (Zhou et al., 2017).

In brief, the identification and classification of adipocyte lineage cells seems simpler than that of MSCs and SSCs at present. More attention should be given to functional studies in the future.

### Linkage Between Fat and Bone

#### Regulation of Differentiation Fate

**FIGURE 3:**
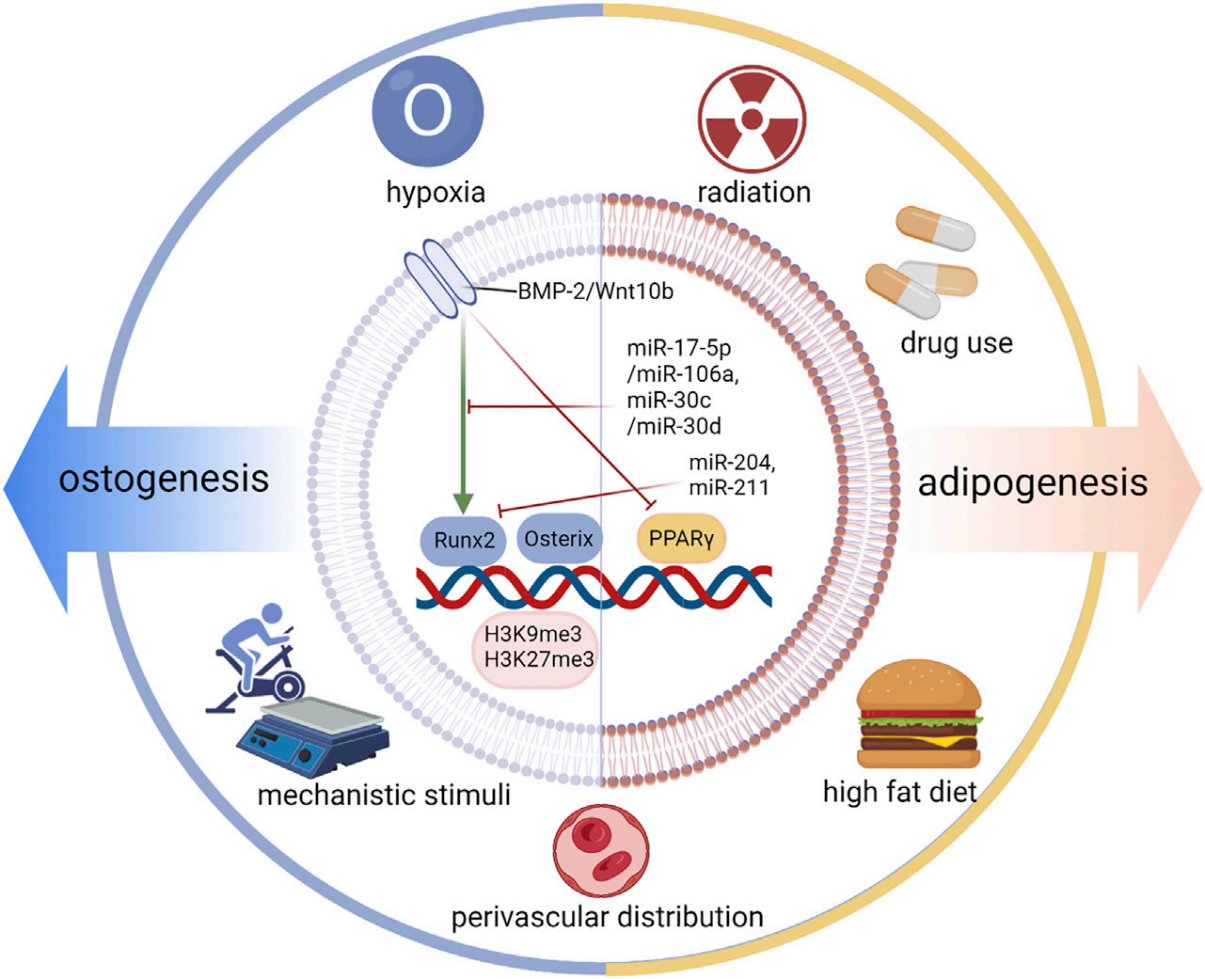
Regulation of differentiation fate. The differentiation fate of BMSCs is specifically regulated by an increase in intracellular transcription factors (TFs), Osterix and PPARγ; signaling pathways; and microRNAs. In addition, extracellular elements such as hypoxia and mechanical stimulation are also involved in this vital process. Runx2, Runt-related transcription factor 2; BMP2, bone morphogenetic protein-2.

As illustrated in many studies, the differentiation fate of BMSCs to osteo-lineage cells and to adipo-lineage bone cells is located on two branches. The differentiation fate is specifically regulated by an increase in intracellular transcription factors (TFs), signaling pathways, and microRNAs. In addition, extracellular elements such as hypoxia and mechanical stimulation are also involved in this vital process (Figure 3).

For osteogenesis, TFs regulate the differentiation fate of BMSCs by increasing the expression of genes that are responsible for the corresponding osteo-cell type. Runt-related transcription factor 2 (Runx2) and Osterix are two key TFs that promote osteoblast differentiation (Augello and De Bari, 2010). Runx2 enhanced by core-binding factor beta (Cbfβ) promotes osteoblast differentiation (Yoshida et al., 2002), but inhibits adipocyte differentiation of BMSCs by disturbing PPARγ (Komori, 2006). Runx2 is also called core binding factor α1 (Cbfa1) and is upregulated in BMSCs through single-cell sequencing (Wolock et al., 2019). Many other factors can increase the TF level of Runx2 and then cooperate with it, such as bone morphogenetic protein-2 (BMP2), Dlx5, Sprouty 2 (Spry2), Twist-1, and Twist-2 (Gori et al., 1999; Bialek et al., 2004; Kronenberg, 2004; Augello and De Bari, 2010; Schneider et al., 2017). Under pathological conditions, unexpected upregulation of Runx2 causes heterotopic ossification (Wang et al., 2019). BMP2 is proven to be a key signaling pathway by targeting Runx2/Cbfa1. High concentrations of BMP2 show dose-dependent effects on osteogenesis (Javed et al., 2008). Furthermore, microRNAs are another important coeffector in this process. MiR-204 and miR-211 are induced during adipogenesis and downregulate Runx2 expression (Huang et al., 2010). MiR-17-5p/miR-106a and miR-30c/miR-30 days inhibit BMP signaling by targeting key components of the pathway, namely, BMP2 and Smad1 (Kang and Hata, 2015). In addition to runx2, a vital factor related to many important signaling pathways and microRNAs, osterix is another primary TF for the osteogenesis of BMSCs. Osterix has been demonstrated to play a role downstream of runx2 and can be activated by runx2 (Nakashima et al., 2002). In osterix-null mice, MSCs cannot differentiate into osteoblasts, and no bone formation occurs (Nakashima et al., 2002). Osterix, also called Sp7, is upregulated in OBs and pre-OBs based on single-cell sequencing (Wolock et al., 2019; Zhong et al., 2020). miR-637 and miR-31 can directly suppress osterix expression (Zhang et al., 2011; Baglìo et al., 2013). In addition to Runx2 and osterix, other transcription factors, such as TAZ and Forkhead box C2 (Foxc2), can also promote osteogenesis and suppress adipogenesis (Hong et al., 2005; Park et al., 2011; Yu et al., 2018). The expression of Wnt10b signaling promotes osteogenesis by inducing the expression of runx2, osterix, distal-less homeobox 5 (Dlx5), and TAZ and suppresses adipogenesis by inhibiting PPARγ and C/EBPα (Bennett et al., 2005; Byun et al., 2014), which demonstrates the pro-osteoblastic and anti-adipocytic differentiation effect of Wnt/β-catenin signaling (Kang and Hata, 2015; Gu et al., 2016). The regulatory function of H3K9me3 and H3K27me3 works in balancing the osteogenic and adipogenic differentiation of mesenchymal stem cells. Ye et al. reported that the histone demethylase KDM4B in BMSCs increases bone marrow fat cells by epigenetic coordination of β-catenin/Smad1-mediated transcription by removing inhibitory H3K9me3, ultimately contributing to bone aging and osteoporosis (Ye et al., 2012). Xue et al. reported that H3K9me3 can activate Wnt-5a and repress PPAR-γ (Xu et al., 2016).

For adipogenesis, PPARγ plays a vital role by regulating the expression of adipogenic genes. PPARγ shows pro-adipocytic and anti-osteoblastic effects (Zhuang et al., 2016). Upstream of FOXO1, it could regulate lipogenesis through PPARγ and the adipocyte cell cycle through p21 and p27 (Chen et al., 2019). PPARγ agonists induce adipocyte differentiation by modulating the expression of Lipin-1 downstream (Kim et al., 2016). PPARγ is a secretory BMP inhibitor (Gustafson et al., 2015). As mentioned above, a high concentration of BMP2 accelerates osteoblast differentiation, while a low concentration of BMP2 promotes adipocyte formation in the C3H10T1/2 mesenchymal cell line (Tang et al., 2004). Additionally, CCAAT/enhancer binding protein α (C/EBPα), platelet-derived growth factor receptor β (PDGFβ) and zinc finger proteins 423 and 521 also take part in adipogenesis (Lin and Lane, 1994; Gupta et al., 2012; Huang et al., 2012; Dang et al., 2021).

In addition to transcription factors, signaling pathways, miRNAs, and other intracellular influencing factors, there are extracellular factors that can also play an important regulatory role, typically including hypoxia, mechanistic stimuli, radiation, a high-fat diet, drug use, and perivascular distribution.

Hypoxia promotes osteogenesis but suppresses adipogenesis of human mesenchymal stromal cells in a hypoxia-inducible factor-1 (HIF-1)-dependent manner (Wagegg et al., 2012). Hypoxia and hypoxia-mimetic microRNA miR-675-5p mediate the angiogenesis response and osteochondroblast commitment of hMSCs (Costa et al., 2017; Wang et al., 2007). Clinically, an FDA-approved iron chelator promotes angiogenesis and osteogenesis, thereby enhancing the rate of fracture repair (Yellowley and Genetos, 2019), while hypoxia promotes osteogenesis but suppresses adipogenesis of human mesenchymal stromal cells in a hypoxia-inducible factor-1 (HIF-1) dependent manner (Wagegg et al., 2012). Hypoxia and hypoxia-mimetic microRNA miR-675-5p in angiogenesis response and osteo-chondroblast commitment of hMSCs (Costa et al., 2017; Wang et al., 2007). Clinically, an FDA-approved iron chelator promotes angiogenesis and osteogenesis, thereby enhancing the rate of fracture repair (Yellowley and Genetos, 2019). Mechanical factors, such as exercise and vibration, have been confirmed by many studies to regulate the differentiation fate of BMSCs. Climbing exercise was reported to significantly increase bone volume and OB number while decreasing bone marrow fat volume and adipocyte number (Mori et al., 2003; Menuki et al., 2008). *In vivo* studies have demonstrated that vibrations with low-magnitude mechanical signals upregulate Runx2 and downregulate PPARγ (Luu et al., 2009). In addition, low-magnitude high-frequency vibration may promote osteoblast differentiation of MSCs *via* the Wnt/β-catenin signaling pathway, the estrogen receptor α signaling pathway, and cytoskeletal remodeling (Haffner-Luntzer et al., 2018; Wang et al., 2020; Yi et al., 2020) (Wang et al., 2020a; Haffner-Luntzer et al., 2018; Yi et al., 2020).

In addition to mechanical stimuli, other chemical factors, such as a high-fat diet and drug use, also play roles in regulating MSC differentiation. Obesity caused by a high-fat diet and aging impair osteogenesis and hematopoietic regeneration by regulating osteoblastic or adipocytic genes, probably through PPAR-γ (Parhami et al., 2001; da Silva et al., 2016; Ambrosi et al., 2017). Yue et al. reported that in diabetes and obesity, LepR signaling in BMSCs has been shown to promote adipogenesis and inhibit osteoblast production in response to diet (Yue et al., 2016). In addition, both obesity and osteoporosis are associated with elevated oxidative stress and increased production of proinflammatory cytokines. The expression levels of DLL1/delta-like one and DLL4/delta-like four ligands decreased under stress conditions. In the absence of vascular Dll4, hematopoietic stem cells prematurely induce bone marrow transcriptional programming (Tikhonova et al., 2019). Deacetylated histone 3 (Hdac3) inhibits lipid storage in osteoblasts and controls fat production (Pierce et al., 2019). Regarding drug use, long-term use of steroid hormones, such as glucocorticoids, can lead to obesity with rapid bone loss (Body, 2010).

In addition, previous studies have suggested that the differentiation directions appear to be correlated with distinguishing the perivascular distribution of cells. Perivascular multipotent stem cells fall into two categories based on the differential expression of the accepted adipose precursor marker stem cell antigen (Sca) 1+ (Ambrosi et al., 2017): around arterioles (PDGFRα+Sca-1-CD45-Ter119- cells) (Morikawa et al., 2009; Ambrosi et al., 2017) and around sinusoids [PDGFRα+Sca-1-CD45-Ter119-, as well as CAR cells (Greenbaum et al., 2013)]. Osteolectin is an osteogenic growth factor that enhances the maintenance of adult skeletal bone cells. It has also been reported that a group of peri-arteriolar LepR+Oln+ cells express osteogenic genes by gene set enrichment analysis. However, LepR+Oln- cells were distributed around the venous sinus and expressed lipogenic genes (Shen et al., 2021).

Taken together, internal factors, such as epigenetic regulation and perivascular distribution, and external factors, such as exercise, drug use, and a high-fat diet, are also critical in regulating osteogenesis and adipogenesis of BMSCs. These factors may take effect through cross-talk with the key transcription factors and signaling pathways mentioned above.

In brief, the differentiation fate regulation of BMSCs presents a complex network.

#### Cross-Talk Between Bone and Fat

Bone growth is the coupling of bone formation by osteoblasts and bone absorption by osteoclasts. In childhood, bone growth is dominated by osteogenesis. During aging, cross-talk occurs between bone and fat. Osteogenesis weakens, bone absorption increases, and fat cells in the bone marrow increase.

Fat is negatively related to bone mass (Shen et al., 2007; Shen et al., 2014). Fat is reported to inhibit bone formation and fracture healing (Ambrosi et al., 2017). Fat can affect bone growth through two or more mechanisms. It was reported that adipo-lineage progenitors specifically and highly express osteoclast regulatory factors. RANKL is the most widely studied one. MALPs can specifically secrete several osteoclast regulatory factors, especially RANKL, which is involved in the progression of bone remodeling (Robling and Bonewald, 2020; Hu et al., 2021; Yu et al., 2021). Adipokines, such as leptin, adiponectin, and chemerin, are typically secreted by adipocytes and are directly or indirectly involved in bone metabolism and correlated with bone mineral density (Fang and Judd, 2018; Helfer and Wu, 2018; Reid et al., 2018; Maeda et al., 2020; Zhao et al., 2020; Fang and Judd, 2018; Helfer and Wu, 2018; Reid et al., 2018; Maeda et al., 2020; Zhao et al., 2020). In pathological situations, such as postmenopausal osteoporosis, obesity has long been considered a beneficial factor for bone health (Felson et al., 1993) that can reduce the risk of fracture (Compston et al., 2014). However, as soon as fracture occurs, the opposite condition occurs. Adipocytic cells significantly impair bone fracture healing and hematopoietic repopulation by secreted dipeptidyl peptidase-4 (DPP4), an important target of antidiabetes treatments (Ambrosi et al., 2017). This adipocyte ablation-mediated enhancement of bone mass reflects the activation of BMP receptors after the elimination of its inhibitor, which is associated with simultaneous epidermal growth factor receptor signaling. Diphtheria toxin receptor adiponectin-induced osteosclerosis was not due to ablation of surrounding fat cells but may reflect the elimination of cells expressing adiponectin in the bone marrow (Zou et al., 2020). To our knowledge, acute fat loss through dieting does not affect bone mass (Lagerquist et al., 2021).

Regarding the effect of bone on fat, there are few studies. However, in many pathological conditions, such as osteoporosis, the phenomenon of expanding the volume and number of adipocytes in BM is generally observed (Justesen et al., 2001). Bone loss is often regarded as a consequence but not an impact factor. The clinical influence of bone on fat needs to be further explored. What is more, many other well-known ways are able to restrain the fat accumulation so that there may be no need to regulate fat through bone and fat cross-talk.

As shown above, cross-talk exists between differentiated bone tissue and fat. The balance between these two is tightly related to physiological homeostasis and pathological situations.

## Conclusion

In conclusion, BMSCs are highly heterogeneous. Different researchers have defined subsets of markers or combinations that are discrete or partially overlapping. Otherwise, those single-cell sequencing studies report their classic and newly discovered roles and connections in the context of physiological or pathological states. The diversity and assortative nature of different cell markers makes it difficult to classify BMSCs, let alone find associations between them. We tried our best to review the identification and classification and to find a link among the overlapping clues of multipotent stem cells, skeletal stem cells, and adipocyte lineage cells for more applicable and clinical explanations.
